# Functionality in Middle-Aged and Older Overweight and Obese Individuals with Knee Osteoarthritis

**DOI:** 10.3390/healthcare6030074

**Published:** 2018-07-04

**Authors:** Neda S. Akhavan, Lauren Ormsbee, Sarah A. Johnson, Kelli S. George, Elizabeth M. Foley, Marcus L. Elam, Zahra Ezzat-Zadeh, Lynn B. Panton, Bahram H. Arjmandi

**Affiliations:** 1 Department of Nutrition, Food and Exercise Sciences, Florida State University, Tallahassee, FL 32306-4310, USA; nsa08@my.fsu.edu (N.S.A.); lormsbee@fsu.edu (L.O.); ksg15c@my.fsu.edu (K.S.G.); ef15c@my.fsu.edu (E.M.F.); ze09@fsu.edu (Z.E.-Z.); lpanton@admin.fsu.edu (L.B.P.); 2 Center for Advancing Exercise and Nutrition Research on Aging (CAENRA), College of Human Sciences, Florida State University, Tallahassee, FL 32306-4310, USA; 3 Department of Food Science and Human Nutrition, Colorado State University, Fort Collins, CO 80523-1571, USA; Sarah.Johnson@colostate.edu; 4 Department of Human Nutrition and Food Science, California State Polytechnic University, Pomona, CA 91768-2557, USA; mlelam@cpp.edu

**Keywords:** pain, joint, body mass index (BMI), men, women, Western Ontario McMaster Universities Arthritis Index (WOMAC), exercise

## Abstract

Patients with knee osteoarthritis (OA) suffer from immobility and pain. The objective of this cross-sectional study was to investigate the relationship between pain and functionality in middle-aged and older overweight and obese individuals with mild-to-moderate knee OA. Overall pattern, physical activity, and total energy expenditure (TEE) were assessed in 83 participants. The Western Ontario McMaster Universities Arthritis Index (WOMAC) was used to assess lower extremity pain and function. The six-minute walk test (6-MWT) and range of motion (ROM) were also assessed. Results indicated that age was inversely associated with body mass index (BMI) (*r* = 0.349) and total WOMAC scores (*r* = 0.247). BMI was positively associated with TEE (*r* = 0.430) and WOMAC scores (*r* = 0.268), while ROM was positively associated with the 6-MWT (*r* = 0.561) and negatively associated with WOMAC (*r* = 0.338) and pain scores (*r* = 0.222). Furthermore, women had significantly greater WOMAC scores (*p =* 0.046) than men. Older participants (≥65 years old) had significantly lower BMI (*p =* 0.002), and distance traveled during the 6-MWT (*p =* 0.013). Our findings indicate that older individuals in this population with knee OA had lower BMI, greater ROM, and less pain and stiffness and walked slower than middle-aged individuals. Women reported greater pain, stiffness, and reduced functionality, indicating that the manifestation of OA may vary due to gender.

## 1. Introduction

Osteoarthritis (OA) is the most common joint disorder in the world and one of the leading causes of disability worldwide [1]. According to reports by the United Nations, 15% of the world’s population over the age of 60 years will have symptomatic OA by the year 2050 [2]. This is also true of developed countries, including the United States of America (US). It is estimated that 72 million Americans are at or approaching retirement age. As the US population increases in age, the prevalence of musculoskeletal conditions associated with functional disability and pain is increasing [3]. Over 20 million Americans have knee OA, a progressive degenerative disease, and suffer from complications including immobility and joint pain [4]. The prevalence is expected to parallel increases in the aging population. OA is characterized by the breakdown of articular cartilage in a synovial joint coupled with the thickening and remodeling of adjacent bone, as well as the growth of osteophytes and bone spurs [5]. OA can also be characterized as a chronic inflammatory disease which affects the whole joint, damaging cartilage and remodeling the subchondral bone structure [6]. Although the symptomatic results of OA are relatively known, the causes of OA are not well established. Quality of life is a major concern for individuals with OA as 80% of individuals with OA have some degree of restriction to their daily activities and 25% suffer from major immobility [7]. The annual costs of care for individuals with OA in the US is estimated to be anywhere between $15 and $26 billion per year, and therefore represents a large economic burden. There is no cure for OA; rather, treatment focuses on delaying the progression of the disease and symptom management [8].

A major modifiable risk factor for the development of OA is excess body weight [9,10,11,12]. Overweight and obese individuals have a greater prevalence of knee OA in comparison with normal weight individuals due, in part, to cartilage degeneration caused by excess body weight [9]. In fact, individuals with a body mass index (BMI) greater than 30.0 kg/m^2^ are 6.8 times more likely to develop knee OA compared to men and women of normal weight BMI between 18.5 and 24.9 kg/m^2^ [9]. Overweight and obese individuals with OA suffer from structural damage of the joints due to increased mechanical load, decreased muscle strength, as well as metabolic changes and inflammation [10]. Body composition is of particular importance in overweight and obese individuals with OA where a 1 kg increase in fat mass (androidal, trunk, or total body) is associated with an increase in tibiofemoral cartilage degeneration [11]. Adipose tissue is a metabolic endocrine organ which can secrete adipokines that can promote inflammation and are detrimental in individuals with OA [12]. Thus, a state of increased inflammation and increased mechanical loading due to obesity may exacerbate pain and stiffness in individuals with knee OA.

The risk for development of OA increases with age; older individuals are at a greater risk for progression of OA due to musculoskeletal changes commonly observed in this population contributing to alterations outside and within the joints [13]. OA typically becomes symptomatic in many individuals after the age of 50 years, possibly due to increases in age-related changes such as increased systemic inflammation, sarcopenia, reduced physical activity, and increased joint stiffness [14]. Particularly in individuals over the age of 65 years, quality of life can be compromised in individuals with OA, and postmenopausal women may manifest, present, and develop the disease differently than men due to anatomic, genetic, and hormonal differences, due to the loss of estrogen [15]. In a meta-analysis by Srikanth et al. [16], women over the age of 55 years tended to have more severe OA particularly in the knee as opposed to other sites in comparison to men, in addition to greater prevalence and incidence of OA.

In addition to pain and inflammation, functionality is a serious problem in individuals with OA and assessment of joint functionality is critical to management and treatment of patients with OA. Reduced range of motion (ROM) and impaired joint mobility are major indicators of disability in individuals with knee or hip OA [17]. The Western Ontario McMaster Universities Arthritis Index (WOMAC) is a widely used clinical questionnaire for assessing the state of individuals with OA, which includes subsections examining pain, stiffness, and physical functioning of the joints [18]. Due to either over- or under-estimation of self-reported functional capacity, objective measures of functionality such as the six-minute walk test (6-MWT) are used to evaluate exercise capacity as well as morbidity and mortality in obese and diseased populations [19]. Establishing the association between knee functionality and disability in this population can provide insight for the assessment as well as the management of symptoms associated with OA.

The exact etiology of OA is unknown and there is a paucity of research examining functionality in overweight and obese individuals who have mild-to-moderate knee OA. Investigating the relationship between pain and functionality in middle-aged and older, overweight and obese men and women with mild-to-moderate knee OA provides insight into potential interventions for reducing the progression and symptoms of OA within this population. Thus, the hypothesis of this cross-sectional study was that BMI would be positively associated with WOMAC, total energy expenditure (TEE), 6-MWT, and pain scores, and negatively associated with ROM and overall functionality measured from the WOMAC in overweight and obese individuals with mild-to-moderate knee OA. We also hypothesized that women and older individuals in the study would have greater WOMAC and pain scores, while having reduced ROM and distance traveled from the 6-MWT.

## 2. Materials and Methods

### 2.1. Participants

Overweight or obese (BMI ≥ 25.0 kg/m^2^), otherwise healthy men and women between the ages of 40 and 90 years with clinically diagnosed mild-to-moderate bilateral or unilateral OA of the knee were recruited for screening from the greater Tallahassee, Florida, and surrounding areas using flyers, radio, and online listings. A Kellgren–Lawrence radiological score of 1–3 was used for diagnosis of knee OA from previous medical records. Participants were excluded if they had a history of severe liver and/or kidney disease or any other chronic or acute diseases that may affect OA, knee surgery (including arthroscopy) or significant injury of the target knee joint within 6 months prior to the start of the study, and any hyaluronan or cortisone injections within 2 months prior to study enrollment. This study was approved by the Florida State University Institutional Review Board. After initial pre-screening over the telephone, eligible participants were asked to come to the Human Performance Laboratory in the Department of Nutrition, Food and Exercise Sciences at Florida State University for an onsite screening and to provide written informed consent. Included participants were able to walk unassisted and had experienced knee pain for at least six months.

### 2.2. Study Overview

After the screening visit and enrollment, participants came for a subsequent visit to the Human Performance Laboratory in the morning seven days later. Basic anthropometric assessments were performed including measurement of height and weight to calculate BMI. Physical activity recalls [20] were performed to assess participants’ physical activity patterns and TEE. ROM, WOMAC, pain assessments, and 6-MWT were used to measure function and levels of pain.

### 2.3. Assessment of Physical Activity

TEE and overall trend of physical activity of participants from the past seven days (with weekdays separately calculated from weekend days) were assessed using Five-City Project Physical Activity Recall [20]. The level of physical activity was based on either moderate (3–5 metabolic equivalents [METs], e.g., mopping and brisk walking), hard (5.1–6.9 METs, e.g., scrubbing floors and jogging), or very hard activities (≥7.0 METs, e.g., cross-country skiing and high-impact aerobics). METs were calculated as follows: 24 h − (time spent sleeping + time doing moderate intensity activity + time doing hard intensity activity + time in very hard intensity activity). The weighted METs/h average was taken and converted to kcal/d.

### 2.4. Assessment of Range of Motion, Functionality, and Lower Extremity Pain

Knee ROM (°) was assessed using a computerized isokinetic dynamometer (Biodex, Shirley, NY, USA). The leg that was most affected by OA was used for ROM testing. Participants were seated in an upright position with the test leg secured at 90°, and the dynamometer arm rotor aligned to the lateral side of the knee joint. Each participant was instructed to raise the leg as high as he/she could for a period of approximately 2 s, long enough for a steady ROM value to be reached. Three repetitions were performed, and an average ROM was recorded. The WOMAC assessment form was presented to each participant for measuring lower extremity pain and function [21]. Participants reported subscale scores from 0 (no pain, stiffness, or difficulty) and 1 (mild) to 4 (extreme) for pain, stiffness, and difficulty in performing basic physical tasks during the previous 48 h. A total score (0–100) was then derived from the tallying of subscale scores. Each participant completed a 6-MWT based on a standardized protocol [22]. The participants were instructed to walk as quickly as possible without causing pain or discomfort to their knees for six minutes on a flat surface while wearing comfortable shoes. During testing, research personnel refrained from walking alongside or in front of the participants to avoid influencing their pace. The total distance covered (meters) was measured using a trundle wheel.

### 2.5. Statistical Analysis

Data were analyzed using Pearson-product moment correlations, where degree of relationships between anthropometric assessments, physical activity, TEE, ROM, WOMAC, and the 6-MWT were determined. The Pearson correlation coefficient r was used to examine the relationship between variables. The magnitudes of correlation coefficients were considered as very small to none (*r* < 0.1), small (0.1 ≤ *r* < 0.3), moderate (0.3 ≤ *r* < 0.5), large (0.5 ≤ *r* < 0.7), very large (0.7 ≤ *r* < 0.9), nearly perfect (0.9 ≤ *r* < 1.0), and perfect (*r* = 1.0) [23]. Independent-sample t-tests were performed to determine if there were differences within this population in BMI, WOMAC, pain scores, ROM, and 6-MWT between males and females as well as older (≥65 years of age) and middle-aged individuals. Statistical analyses were performed with the IBM (International Business Machines) SPSS (Statistical Package for the Social Sciences) computer program (version 22). All data are presented using means ± standard deviation. All significance was accepted at *p* ≤ 0.05.

## 3. Results

**Figure 1:**
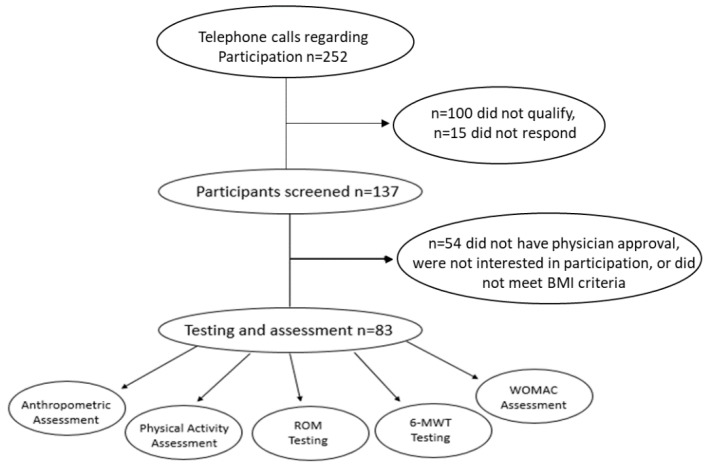
Study flowchart.

A total of 137 participants were determined to be eligible for an in-person screening (Figure 1) through an initial telephone screening of 252 participants. From the 252 participants who were telephone screened, 100 participants did not meet the inclusion criteria and 15 did not return calls. From the 137 participants who were screened on-site, there were 83 participants, 66 females and 17 males who met the age inclusion criteria, that were diagnosed with mild-to-moderate bilateral or unilateral OA of the knee and a Kellgren–Lawrence radiological score of 1–3 who met the inclusion criteria and were enrolled in this cross-sectional study. Common causes for participant exclusion were non-physician approval of mild-to-moderate knee OA, not meeting the BMI criteria (BMI ≤ 24.9 kg/m^2^), traveling, and not being interested in participating in the study.

### 3.1. Baseline Characteristics

The mean age of participants was 62.0 ± 9.2 years old with an average height of 167.0 ± 8.4 cm, weight of 90.0 ± 19.5 kg, and BMI of 32.5 ± 6.2 kg/m^2^ (Table 1). The mean for ROM, 6-MWT, and WOMAC scores were 68.2 ± 13.4°, 407.6 ± 88.1 m, and 53.4 ± 15.4, respectively (Table 1). The mean number of hours per day for moderate, hard, and very hard activity was 2.78 ± 2.74, 1.07 ± 0.52, and 0.13 ± 0.04 h/day, respectively, with a mean TEE of 3809 ± 1120 kcals/day (Table 1).

### 3.2. Age

**Figure 2:**
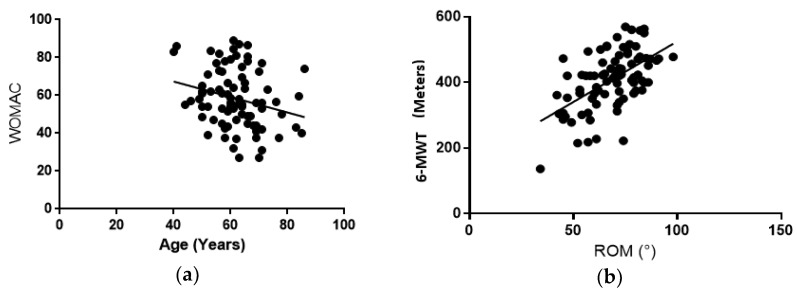
Right panel (**a**): WOMAC score vs. age. As participants aged, WOMAC scores decreased, (*r* = −0.247, *p* = 0.025). Left panel (**b**): 6-MWT vs. ROM. Regardless of age or gender, those who were able to walk further during the 6-MWT had a greater knee ROM (*r* = 0.561, *p* < 0.001).

Significant negative correlations were found between age and weight (*r =* −0.299, *p* = 0.006), BMI (*r =* −0.349, *p* = 0.001), and total WOMAC scores (*r =* −0.247, *p* = 0.025) (Figure 2). These results indicate that as age increases, body weight decreases and knee functionality increases.

Older participants (≥65 years old) had significantly lower BMI (*p* = 0.002), and less distance traveled during the 6-MWT (*p* = 0.013) in comparison to middle-aged participants (<65 years old) who had no significant differences in ROM (*p* = 0.118) and WOMAC scores (*p* = 0.091) (Table 2).

### 3.3. Body Mass Index (BMI)

BMI was positively correlated with TEE (*r =* 0.430, *p* < 0.001 and WOMAC scores (*r =* 0.268, *p* = 0.014). This indicates that higher BMI’s are associated with increased energy expenditure and decreased functionality.

### 3.4. Range of Motion (ROM)

**Figure 3:**
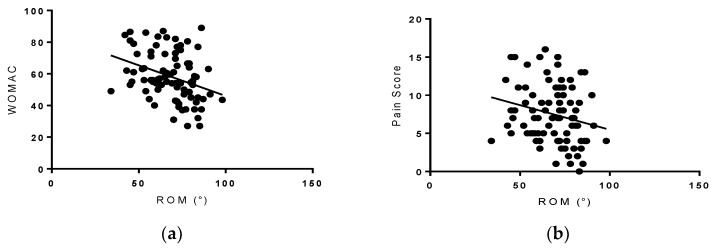
Right panel (**a**): WOMAC score vs. ROM. Individuals with a lower WOMAC score had greater knee ROM (*r* = −0.338, *p* = 0.002). Left panel (**b**): pain score vs. ROM. Individuals with a lower pain score had a greater knee ROM (*r* = −0.222, *p* = 0.002).

**Figure 4:**
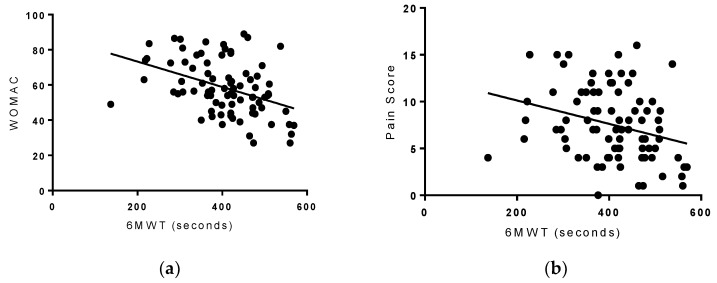
Right panel (**a**): WOMAC score vs. 6-MWT. While WOMAC scores decreased, distance walked during the 6-MWT increased (*r* = −0.413, *p* < 0.001). Left panel (**b**): pain score vs. 6-MWT. As pain scores decreased, distance walked during the 6-MWT increased (*r* = −0.222, *p* = 0.010).

ROM was positively associated with the 6-MWT (*r =* 0.561, *p* < 0.001 (Figure 3) and negatively associated with WOMAC (*r =* −0.338, and *p* = 0.002) and pain scores (*r =* −0.222, *p* = 0.002) (Figure 4).

### 3.5. Six-Minute Walk Test (6-MWT)

The 6-MWT negatively correlated with WOMAC scores (*r =* −0.413, *p* < 0.001 and pain scores (*r =* −0.222, *p* = 0.010) (Figure 4), while positively correlating with very hard activity (*r =* 0.238, *p* = 0.030). These results indicate that participants who were able to go a longer distance with the 6-MWT, tended to have lower pain and higher functionality scores.

### 3.6. Total Energy Expenditure (TEE)

TEE was positively associated with weight (*r =* 0.476, *p* < 0.001, moderate activity (*r =* 0.567, *p* < 0.001, and hard activity (*r =* 0.544, *p* < 0.001 (Table 3).

### 3.7. Gender

Women had significantly greater WOMAC scores (*p* = 0.046) than men with no significant differences in pain scores (*p* = 0.163), ROM (*p* = 0.930), BMI (*p* = 0.694), and distance traveled during the 6-MWT (*p* = 0.467).

## 4. Discussion

The results of this study demonstrate that increased functionality was associated with lower WOMAC and lower pain scores, which is to be expected since when people have less pain it is easier for them to conduct activities of daily living. The same was also true about the 6-MWT, as individuals with lower WOMAC and pain scores were able to walk longer distances. Additionally, participants who had a greater ROM were able to walk a longer distance with the 6-MWT, and participants who were able to walk longer distances with the 6-MWT, on average, participated in more very hard activities. Although there are numerous studies [24,25,26,27,28,29,30] which have performed one or two tests in this population, the authors are not aware of any studies that have examined WOMAC pain scores, ROM, and 6-MWT simultaneously. These findings confirm that it is most likely that once people suffer from pain due to OA, their overall functionality is also compromised. Older age was associated with a lower BMI, WOMAC scores, and shorter distance traveled from the 6-MWT, while increases in BMI were associated with increases in WOMAC scores and TEE. Women had greater WOMAC scores than men, indicating that gender may play an additional role in the progression and manifestation of OA. Additionally, this may explain our distribution of males and females included in this study, as the prevalence of OA in the knee is higher in women [31]. These findings provide insight into the functional capacity in overweight and obese individuals with mild-to-moderate OA, which is important for maintenance of knee joint function, as well as targets for possible therapies to prevent progression of OA in this population. These results indicate that these individuals may want to quickly complete tasks in order to have decreased pain. The authors were surprised to find that these individuals also participated in more very hard activities, an unexpected finding that may be due to the short duration of these types of activities, allowing individuals to get a given task finished faster, possibly contributing to less pain.

In this study the WOMAC questionnaire was used to assess functionality and pain. The participants who had higher BMI were found to have more pain, stiffness, and loss of physical function. Oyeyemi et al. [24] observed a similar trend with regard to BMI and functional limitations, where obese participants with knee OA had higher WOMAC scores than normal and overweight counterparts. Contrary to our expectations, findings from our study also showed that the older participants had a lower BMI and WOMAC scores. OA is an age-related disorder, where aging can increase wear and tear of joints, increasing pain and disability [25]. Perceived pain in older individuals due to musculoskeletal conditions is very common and often underreported. Another possible explanation for lower WOMAC scores observed in the older participants in this study may possibly be due to successful pain management as well as improvements in overall quality of life [26]. ROM was inversely related to WOMAC scores, indicating that in individuals with mild-to-moderate knee OA, ROM can be a good indication of preserved functionality. In a study by Kauppila et al. [26] there was no significant association between ROM and self-reported disability in individuals with severe knee OA. These contrasting findings may have been due to the severity of knee OA within the population studied, whereas in our study the individuals did not have as severe knee OA. Individuals who were able to travel greater distances from the 6-MWT had significantly lower WOMAC scores. Our results are similar to those of Sutbeyaz et al. [28] who observed a significant negative correlation between the 6-MWT and WOMAC scores in obese individuals with mild to severe knee OA. In regard to gender, women had greater WOMAC scores than men, which is consistent with previous studies indicating that women with a similar degree of knee OA have greater perceived knee pain in comparison to men [29,30].

The participants in this study who had a greater ROM had a lower WOMAC score, and the participants with greater ROM were able to go a longer distance with the 6-MWT. Participants who were able to walk longer distances with the 6-MWT also participated in more very hard activities throughout their days. The older individuals in this study did not travel as far a distance with the 6-MWT than the middle-aged individuals. A study by Nejati et al. [32] found that adding anaerobic exercises to non-invasive therapies for knee OA decreased pain and improved knee function after three months. A meta-analysis performed by Tanaka et al. [33] examining different exercise therapies in patients who have knee OA found that both muscle strengthening and aerobic exercises were effective in reducing pain, with non-weight bearing strengthening exercises being the most effective. While these studies have identified possible treatments useful for improving both quality of life and the aging process, prevention of the disease is still poorly understood.

Several longitudinal and cross-sectional studies from different countries have recognized the strong association between OA, obesity and reduced functionality [34,35,36,37]. The Rotterdam Study, which followed 3585 participants from the Netherlands for over 6 years, observed that a BMI > 27 kg/m^2^ was associated with a 3.3-fold increased risk and progression of knee OA [34]. Similarly in the Chingford Study, 715 women from the London area were followed for 4 years and women who were in the top tertile of body weight (BMI > 26.4 kg/m^2^) had an increased risk of knee OA [35]. The China Health and Retirement Longitudinal Study, which was a population based survey that examined women over the age of 45 years from 3 different provinces in China, observed that the prevalence of knee OA was higher in China than that of Americans (the Framingham Study) as well as Europeans (Epidemiological Study of Rheumatic Diseases in Greece), indicating that in addition to obesity there may be genetic and other lifestyle factors which may play an important role in the etiology of knee OA [36].

Participants who had greater TEE also had a higher BMI, and participated in more moderate and hard activities throughout their days in this study. This is of particular importance within this population because less than one in seven men and one in 12 women who have OA of the knee get enough physical activity [37]. A study by Christensen et al. [38] showed that 10% weight loss in obese individuals with knee OA resulted in improvement in functionality by 28%. Thus, increases in TEE may be an optimal component of knee OA management in overweight and obese individuals. As obese individuals with knee OA reduce body weight it may contribute to a reduction in pain and distress associated with movement. A systematic review and meta-analysis from the same group [39] observed that obese adults with knee OA who lost 5% of their body weight had a reduction in joint pain, and when there was a loss of at least 10% of their body weight they had further improvements in joint pain.

Limitations of this study include the calculation of TEE from the Five-City Project questionnaire which is a subjective assessment of physical activity. Many physical activity questionnaires are known to overestimate TEE, particularly in overweight and obese individuals [40,41]. Administration of questionnaires, such as the WOMAC and physical activity recall were subjective measures which, with all self-reports, may have some bias with how it is reported which may have influenced some of our results. Participants in this study were also not homogeneous with regards to gender, with more women being eligible for participation than men. The greater number of female participants may be due to the higher prevalence of OA in women, as well as more women being interested in the study.

## 5. Conclusions

The results from this study indicate that overweight and obese individuals with knee OA had a greater ROM and TEE from physical activity, less pain and stiffness, and walked longer distances on average than those who had lower ROM and higher pain, which was in agreement with our hypothesis. Further studies are needed to establish the relationship between pain and which types of physical activity will preserve functionality in this population.

## Figures and Tables

**Table 1:** Participant characteristics (*n* = 83).

	All (*n* = 83)	Female (*n* = 66)	Male (*n* = 17)	Range
**Age (years)**	62.0 (9.0)	61.2 (8.6)	64.8 (8.8)	52.8–71.2
**Weight (kg)**	90.0 (19.5)	88.6 (20.0)	99.4 (15.7)	70.5–109.5
**Height (cm)**	167.0 (8.4)	164.6 (7.1)	176.5 (6.5)	158.6–175.4
**BMI (kg/m^2^)**	32. (6.2)	32.6 (6.6)	31.9 (4.9)	26.3–38.7
**ROM (°)**	68.2 (13.4)	64.1 (13.9)	68.4 (11.7)	54.8–81.6
**6-MWT (meters)**	407.6 (88.1)	404.1 (87.6)	421.3 (91.3)	319.5–495.7
**WOMAC**	53.4 (15.4)	60.1 (14.9)	51.8 (15.7)	38.0–68.7
**Moderate (hours)**	2.8 (2.8)	2.7 (2.7)	2.8 (3.0)	0.0–5.6
**Hard (hours)**	0.5 (1.0)	0.5 (1.1)	0.7 (0.8)	0.0–1.5
**Very Hard (hours)**	0.04 (0.14)	0.05 (0.15)	0.02 (0.06)	0.0–0.18
**TEE (kcals)**	3809 (1120)	3707 (1105)	4201 (1120)	2688–4929

Values are shown as means and standard deviations (SD). Range of motion (ROM); six-minute walk test (6-MWT); Western Ontario McMaster Universities Arthritis Index (WOMAC); moderate, hard, and very hard physical activity (PA); total energy expenditure (TEE).

**Table 2:** Body mass index (BMI) and physical functionality outcomes (*n* = 83).

	Female (*n* = 66)	Male (*n* = 17)	*p*-Value	<65 years (*n* = 55)	≥65 years (*n* = 28)	*p*-Value
**BMI (kg/m^2^)**	32.9 (6.7)	31.2 (4.8)	*p* > 0.05	43.0 (6.7)	29.6 (4.0) ^t^	*p* = 0.002
**ROM (°)**	64.8 (12.8)	78.1 (9.8)	*p* > 0.05	69.8 (12.6)	65.0(14.6)	*p* > 0.05
**WOMAC**	60.1 (14.9) *	51.8 (15.7)	*p* = 0.046 *	54.4 (15.8)	60.4(14.9) ^t^	*p* > 0.05
**6-MWT**	388.0 (85.8)	465.4 (65.3)	*p* > 0.05	424. 5(84.2)	374.0(87.6) ^t^	*p* = 0.013 ^t^

Values are shown as means and standard deviations (SD). Total WOMAC scores for females were significantly greater than male values. Additionally, BMI, distance traveled for the 6-MWT were significantly lower in older (≥65 years) individuals when compared to those of middle age, regardless of gender. (*) Indicates significant differences between females and males; (^t^) indicates significant differences between individuals <65 years and ≥65 years.

**Table 3:** Pearson product-moment correlations between dependent variables (*n* = 83).

Variables	Age (years)	Height (cm)	Weight (kg)	BMI (kg/m^2^)	ROM (°)	6-MWT (m)	MOD (h)	HARD (h)	VHARD (h)	TEE (kcal)	WOMAC	PAIN
Age (years)	1	−0.007	−0.299 ^a^	−0.349 ^a^	0.006	−0.124	0.002	−0.071	−0.145	−0.2	−0.247 ^a^	−0.378
Height (cm)	−0.007	1	0.45	−0.004	0.067	0.006	−0.193	0.131	−0.076	0.22	−0.184	−0.113
Weight (kg)	−0.299 ^a^	0.45	1	0.888	−0.045	−0.136	−0.125	0.045	−0.148	0.476 ^f,g^	0.145	0.149
BMI (kg/m^2^)	−0.349 ^a^	−0.004	0.888	1	−0.072	−0.151	−0.04	−0.009	−0.135	0.430 ^b^	0.268 ^b^	0.237
ROM (°)	0.006	0.067	−0.045	−0.072	1	0.561 ^c^	0.063	0.139	0.2	0.159	−0.338 ^d^	−0.222 ^d^
6-MWT	−0.124	0.006	−0.136	−0.151	0.561 ^c^	1	0.108	0.096	0.238 ^e^	0.061	−0.413 ^e^	−0.283
MOD (h)	0.002	−0.193	−0.125	−0.04	0.063	0.108	1	0.236	0.055	0.567 ^g^	0.03	0.09
HARD (h)	−0.071	0.131	0.045	−0.009	0.139	0.096	0.236	1	0.148	0.544 ^g^	0.058	0.114
VHARD (h)	−0.145	−0.076	−0.148	−0.135	0.2	0.238 ^e^	0.055	0.148	1	0.078	−0.109	−0.014
TEE (kcal)	−0.2	0.22	0.476 ^f,g^	0.430 ^b^	0.159	0.061	0.567 ^g^	0.544 ^g^	0.078	1	0.096	0.174
WOMAC	−0.247 ^a^	−0.184	0.145	0.268 ^b^	−0.338 ^d^	−0.413 ^e^	0.03	0.058	−0.109	0.096	1	0.845
PAIN	−0.378	−0.113	0.149	0.237	−0.222 d^e^	−0.283	0.09	0.114	−0.014	0.174	0.845	1

^a^ Significant (−) correlations were found between age and weight, BMI, and total WOMAC, an indicator that as age increases, body weight and knee functionality decline; ^b^ Significant (+) correlations were found between BMI and TEE, WOMAC, indicating that higher BMI scores are associated with increased TEE and a decline in functionality; Significant (+) ^c^ and (−) ^d^ correlations were found between ROM and 6-MWT and with ROM and WOMAC pain scores, respectively. Increased ROM along with a longer 6-MWT was expected as well as a decrease in WOMAC since pain was lower as well. ^e^ 6-MWT was significantly (−) correlated with WOMAC and pain (pain not significant) while (+) correlating with very hard activity, suggesting lower pain/higher functionality with longer 6-MWT. ^g^ Significant (+) associations were found between TEE and weight, moderate, and hard activity.
